# Evidence of superficial knowledge regarding antibiotics and their use: Results of two cross-sectional surveys in an urban informal settlement in Kenya

**DOI:** 10.1371/journal.pone.0185827

**Published:** 2017-10-02

**Authors:** Sylvia Omulo, Samuel M. Thumbi, Svetlana Lockwood, Jennifer R. Verani, Godfrey Bigogo, Geoffrey Masyongo, Douglas R. Call

**Affiliations:** 1 Paul G. Allen School for Global Animal Health, Washington State University, Pullman, WA, United States of America; 2 Community Health Analytics Initiative, Washington State University, Pullman, WA, United States of America; 3 Center for Global Health Research, Kenya Medical Research Institute, Kisumu, Kenya; 4 Centers for Disease Control and Prevention (CDC), Atlanta, GA, United States of America; 5 The Nelson Mandela African Institute for Science and Technology, Arusha, Tanzania; Imperial College London, UNITED KINGDOM

## Abstract

We assessed knowledge and practices related to antibiotic use in Kibera, an urban informal settlement in Kenya. Surveys was employed at the beginning (entry) and again at the end (exit) of a 5-month longitudinal study of AMR. Two-hundred households were interviewed at entry, of which 149 were also interviewed at exit. The majority (>65%) of respondents in both surveys could name at least one antibiotic, with amoxicillin and cotrimoxazole jointly accounting for 85% and 77% of antibiotics mentioned during entry and exit, respectively. More than 80% of respondents felt antibiotics should not be shared or discontinued following the alleviation of symptoms. Nevertheless, 66% and 74% of respondents considered antibiotics effective for treating colds and flu in the entry and exit surveys, respectively. There was a high (87%, entry; 70% exit) level of reported antibiotic use (past 12 months) mainly for colds/flu, coughs and fever, with >80% of respondents obtaining antibiotics from health facilities and pharmacies. Less than half of respondents remembered getting information on the correct use of antibiotics, although 100% of those who did reported improved attitudes towards antibiotic use. Clinicians and community pharmacists were highly trusted information sources. Paired household responses (n = 149) generally showed improved knowledge and attitudes by the exit survey although practices were largely unchanged. Weak agreement (κ = -0.003 to 0.22) between survey responses suggest both that unintended learning had not occurred, and that participant responses were not based on established knowledge or behaviors. Targeted public education regarding antibiotics is needed to address this gap.

## Introduction

Antibiotic use practices, which impact antimicrobial resistance (AMR) worldwide, can vary among individuals, populations and regions [1,2]. This variation is governed by multiple factors including access to- and quality of antibiotics [3], prevalence and types of diseases in a population, nature and quality of health services [1], level of public health education [4] and the political landscape of a region [2]. Despite this complexity, unregulated antibiotic use is the most commonly cited factor contributing to AMR, particularly in low- and middle-income countries (LMICs) [5].

Successful control of AMR depends, in part, on understanding the human factors that underlie the use of antibiotics. Knowledge, attitude and practice (KAP) surveys commonly aim to identify factors that influence behaviors, and can form the basis for population-specific interventions [6]. Unfortunately, few KAP surveys on antibiotic use and related factors have been reported in sub-Saharan Africa where disease burdens can be high and consequently elevate the demand for antibiotics. Even where these surveys are conducted, informal settlements (slums) have rarely been the focus. Informal settlements are complex areas that are characterized by high population densities, poor housing and sanitation infrastructure, high disease burden and limited healthcare facilities [7–9]. Often, they present a mix of factors that can perpetuate the generation and transmission of resistant bacteria. With the global population residing in informal settlements projected to reach two billion within the next thirty years [10], the importance of AMR intervention efforts in these communities should be emphasized.

The goal of this study was to assess knowledge about antibiotics and antibiotic use practices among people living within an urban informal settlement in Kenya. Surveys were carried out at the beginning and end of a five-month longitudinal observational study focused on antimicrobial resistance. As a secondary objective, we evaluated whether “unintended learning” about antibiotics and their use had occurred among the surveyed respondents, owing to their voluntary participation in the longitudinal study.

## Methods

### Study population

This study was based in Kibera; a large, densely-populated informal settlement situated within Nairobi, a city of >3 million inhabitants [8]. Surveys were conducted in two “villages,” Soweto and Gatwekera, which have been the site for a population-based infectious disease surveillance (PBIDS) [11] carried out by the Kenya Medical Research Institute (KEMRI) and the Centers for Disease Control and Prevention (CDC). PBIDS participants receive free health services for acute illnesses at the Tabitha health clinic, which is located within the study area. Soweto and Gatwekera have a population density of approximately 77,000 people/km^2^ and are characterized by poor sanitation, unregulated water supply systems and outdoor food vending with inconsistent and minimal hygiene [12]. Diarrhea, respiratory and febrile illnesses are prevalent in this area [11–14]. As with many parts of Nairobi where access to antibiotics is largely uncontrolled [15], a variety of pharmacies–both licensed and unlicensed–serve these villages.

### Survey design

Two surveys were conducted; at the beginning (‘entry’, September 2015) and end of a longitudinal study (‘exit’, between Dec 2015 and Jan 2016). From among 5,320 households participating in PBIDS at the time of this study, 200 randomly selected households consented to participate in the longitudinal study on antimicrobial resistance, out of 217 households that were approached to participate. A sample size of 200 provides a 95% confidence interval of 7% for any given parameter estimate. For each selected household, a single adult representative (>18 years) with knowledge of household healthcare practices was invited to enroll into the study and participated in both surveys. Household demographic data, including the age of the respondent, household size and structure, and education levels of the male and female heads of households, were collected during enrollment. Surveys were administered by community interviewers who were trained to recognize commonly-used antibiotics and to administer the structured questionnaire used for this study. This questionnaire (S1 File) addressed three broad topics: a) knowledge of antibiotics, b) sources of antibiotics and reasons for taking antibiotics, and c) sources of information about antibiotics and the impact of this information on antibiotic use attitudes and practices. The absence of a locally-recognizable Swahili term for the word “antibiotic” necessitated a creative approach to conducting the surveys. Interviews were initiated by asking respondents to name up to three medications–excluding pain-relievers, traditional herbs and anti-malarial drugs–that they had used in the past or about which they were conversant. Probing was done until up to three antibiotics were mentioned. Respondents who were unable to recall any antibiotic were not interviewed further. Respondents who mentioned at least one antibiotic were probed with subsequent true-or-false questions (Table 1) that referenced, by name, the first antibiotic mentioned by the respondent (denoted henceforth as “medication X”). Data were checked for accuracy and completeness within a day of collection and compiled in a Microsoft Access database (Microsoft Corp., Redmond, WA).

**Table 1:** Proportion of responses for which respondents responded “FALSE” for a set of true-or-false questions regarding the use of antibiotics.

*Do you think these statements are “TRUE” or “FALSE”?* (Expected response: “FALSE”)		
	Entry (n = 134)	Exit (n = 122)
1. one should stop taking {medication “X”} when one feels better	112 (83.6%)	113 (93.4%)
2. {medication “X”} is effective against colds and flu	50 (34.3%)	31 (25.6%)
3. it is okay to share {medication “X”} with someone else	116 (86.6%)	118 (97.5%)

Figures in parenthesis show the percentage of the total household responses.

### Data analysis

Summaries on antibiotic knowledge and antibiotic use practices were generated in Microsoft Excel (2016). The occurrence of unintended learning about antibiotics and their use was determined by conceptualizing the same individual before and after “exposure” to the longitudinal study as equivalent to a pair of “raters” who are temporally distinct, and by using agreement statistics (Cohen’s kappa) in R (version 3.3.1) [16] to determine if people retained their original responses (high agreement) or changed them (low agreement).

Changes in antibiotic-related knowledge, attitudes and practices were analyzed for households for which data was available for both entry and exit surveys (n = 149). Knowledge changes were assessed based on respondents’ ability to name antibiotics and to correctly respond to the true-or-false questions regarding their use and whether they recalled receiving any information about the correct use of antibiotics. Attitude changes were assessed broadly to not only include the effect of antibiotic-related information on household antibiotic use practices but also dependence on healthcare institutions or healthcare workers for information regarding antibiotics and their use. Practice changes focused on reported behaviors relating to the use of antibiotics, and household decisions regarding the acquisition of antibiotics. Discordant pairs of survey responses by the same respondent were quantified as change. The Wilcoxon’s rank-sum test was used to determine whether changes, if any, were statistically significant (*P* < 0.05).

We further analyzed participant responses to determine their direction of change. For the true-or-false knowledge questions, households in which respondents gave a “TRUE” response at the entry survey and “FALSE” at the exit survey. Questions that had several response options were analyzed for each option separately allowing them to be analyzed as “Yes-No” questions. We considered changes that led to improved knowledge, attitudes or practices as positive changes, otherwise the changes were considered negative. Thus, although information on quality—for example—of community pharmacists was not collected, we quantified attitudes and practices that favored contact with these healthcare sources as positive while those that did not (e.g. reliance on previous experiences or receiving information from family and friends) as negative.

This study was approved by the Kenya Medical Research Institute Scientific and Ethic’s Review Committee (SSC Protocol # 2998), the Centers for Disease Control and Prevention (CDC protocol # 6761) and the Washington State University Institutional Review Board (IRB Number #14413–002). Oral and written consent were also obtained from study respondents before enrollment into the longitudinal study.

## Results

Two-hundred households were interviewed during the entry survey out of which 149 were interviewed during the exit survey (~5 months between entry and exit surveys). The majority (>93%) of respondents in both surveys were female. The mean age of respondents was 28.7 in the entry survey and 29.0 in the exit survey. The median household size was five people (range: 2–13) and all households had at least one child aged ≤5 years. The median level of education attained by male and female household heads was primary school.

### Knowledge of antibiotics

**Fig 1:**
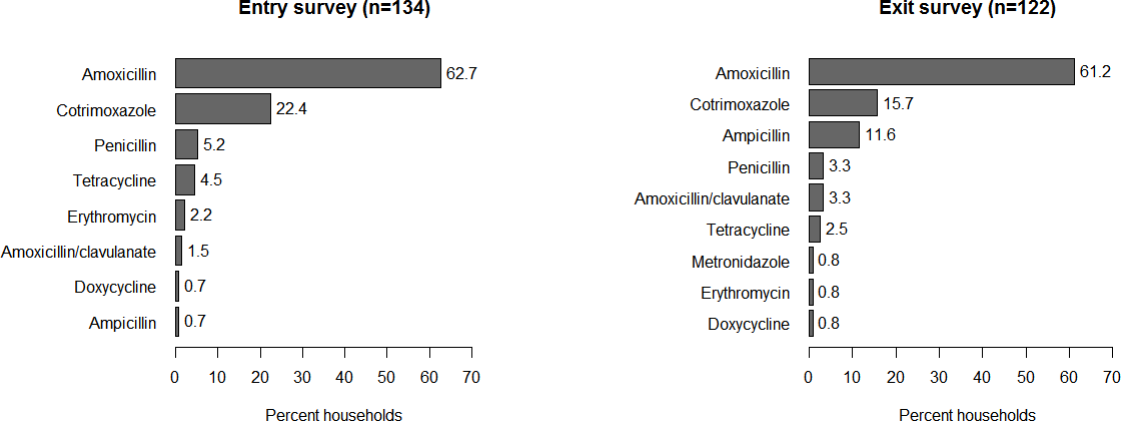
Proportion of household respondents that mentioned an antibiotic* during the entry and exit surveys.

Of the surveyed respondents, 67% named at least one antibiotic in the entry survey and 82% in the exit survey. All the antibiotics mentioned in the entry survey were mentioned in the exit survey, with the exception of metronidazole (exit only; Fig 1). Cotrimoxazole and amoxicillin were the most commonly mentioned antibiotics, jointly accounting for 85% and 77% of all responses in the entry and exit surveys, respectively. Overall, there were no differences in the frequency of respondents that mentioned various antibiotics for both surveys (ANOVA, *P* = 0.29). In both surveys, only 10% mentioned a second antibiotic—commonly amoxicillin or cotrimoxazole—while none mentioned a third antibiotic. The majority (≥ 84%) of respondents in both surveys reported that antibiotic use should not be discontinued following the alleviation of symptoms, and that antibiotics should not be shared. Nevertheless, 66% and 74% of respondents considered antibiotics effective for treating colds and flu in the entry and exit surveys, respectively (Table 1).

### Sources of antibiotics and antibiotic use practices

When asked where they would obtain the self-identified antibiotics when needed, most respondents mentioned health facilities (80%, entry and 69%, exit), and pharmacies (25%, entry and 30%, exit). In both surveys, the few respondents who mentioned penicillin, doxycycline and amoxicillin/clavulanate only named health facilities as potential sources for these antibiotics.

Eighty-seven percent of respondents surveyed during the entry survey reported having used an antibiotic within the 12-month period preceding the study whereas 70% reported previous use in the exit survey. More specifically, 89.3% of the 85 respondents that named amoxicillin and 90% of the 30 respondents that named cotrimoxazole in the entry survey reported having used it in the past 12 months. In the exit survey, 66% of the 47 respondents that named amoxicillin (n = 47), and 68% of the 19 respondents that named cotrimoxazole reporting having used it within the same period. Most (13 out of 14) respondents that mentioned ampicillin in the exit survey reported having used it in the past year (only one respondent mentioned ampicillin in the entry survey).

When asked to name the illnesses for which they would normally decide to take the antibiotics mentioned, respondents mentioned colds/flu, coughs, diarrhea, headache, fever, pneumonia and malaria (Table 2). These were either mentioned as single ailments or as combinations of ailments. Amoxicillin, ampicillin, and cotrimoxazole use were mentioned against all of these conditions. Penicillin was not mentioned in the case of malaria, while tetracycline was not mentioned in the case of malaria and pneumonia. Amoxicillin/clavulanate and doxycycline were only mentioned in the case of colds and coughs while erythromycin was mentioned for colds, coughs, diarrhea and fever. Metronidazole was the only antibiotic that was mentioned against one condition (diarrhea).

**Table 2:** Survey responses to questions regarding antibiotic use, sources of antibiotics and information on antibiotics.

**Have you taken {medication “X”} within the last 12 months?**		
	**Entry (n = 134)**	**Exit (n = 122)**
*Yes*	116 (86.6%)	85 (69.7%)
**In what ways would you obtain {medication “X”} if you felt you needed to use it?**		
*Health facility*	107 (79.9%)	84 (68.9%)
*Chemist*	34 (25.4%)	37 (30.3%)
*Left-over*	1 (0.7%)	7 (5.7%)
*Elsewhere*	1 (0.7%)	5 (4.1%)
**How would you normally know which medicine to buy when someone falls ill?**		
*Recommendation by community pharmacist*	93 (69.4%)	65 (53.7%)
*Previous prescription by a clinician*	39 (29.1%)	38 (31.4%)
*Own experience*	26 (19.4%)	20 (16.5%)
*Opinion of family or friends*	5 (3.7%)	9 (7.4%)
**In the last year, do you remember getting information about proper use of {medication “X”}?**		
*Yes*	35 (31.1%)	56 (45.9%)
**From where did you get information?**		
*Clinician*	25 (71.4%)	32 (57.1%)
*Community pharmacist*	15 (42.9%)	14 (25.0%)
*Other health professionals (e.g. nurse)*	5 (14.3%)	10 (17.9%)
*Friend/family member*	3 (8.6%)	4 (7.1%)
**In what ways did the information change your views about the use of {medication “X”}?**		
*Complete dosage*	16 (45.7%)	25 (44.6%)
*Always consult a clinician*	15 (42.9%)	32 (57.1%)
*Not take this medicine without prescription*	10 (28.6%)	27 (48.2%)
**Which sources would you trust if you needed information on {medication “X”}?**		
*A clinician*	118 (88.1%)	95 (78.5%)
*A community pharmacist*	64 (47.8%)	98 (81.0%)
*A hospital*	40 (29.9%)	55 (45.5%)
*A nurse*	27 (20.1%)	51 (42.1%)
**For which illnesses would you normally decide to take {medication “X”}?**		
*Colds/flu*	81 (60.4%)	57 (46.7%)
*Cough*	81 (60.4%)	60 (53.3%)
*Fever*	53 (39.6%)	23 (18.9%)
*Diarrhea*	21 (15.7%)	6 (4.9%)
*Malaria*	13 (9.7%)	7 (5.7%)

Multiple responses were allowed for survey questions.

The combination of symptoms for which most respondents reported they would use an antibiotic was colds/flu with an accompanying cough (26%, entry and 30%, exit). The presence of fever as an additional symptom accounted for an additional 15% of responses in the entry survey and 5% in the exit survey. Antibiotic use for coughs accounted for 7% of responses in the entry survey and 23% in the exit survey. Of the respondents who named any antibiotic and said that they would take it for colds/flu + cough (n = 30, entry and n = 25, exit), 80% named amoxicillin in the entry survey and 56% in the exit survey. An additional one-third (32%) named cotrimoxazole in the exit survey.

### Antibiotic information sources and their impact on antibiotic usage

Approximately 70% of respondents surveyed at entry and 54% of those surveyed at exit reported that they rely on community pharmacists to recommend medication in the event of an illness within the household. One third of respondents also reported relying on previous prescriptions by a clinician and almost 20% reported that their choice would be based on their own experience. Few respondents reported relying on the opinions of family or friends when deciding which medicine to buy (Table 2).

Less than half of all surveyed respondents (31.1%, entry and 45.9%, exit) recalled getting information regarding proper use of antibiotics in the 12-month period preceding the two surveys. The primary information sources were clinicians and community pharmacists, who were also considered the most trustworthy sources of information regarding antibiotics. In both surveys, all respondents that recalled getting information reported that it changed their views on the usage of the antibiotic. These changes in views included always consulting a clinician, not taking non-prescribed antibiotics and completing antibiotic doses (Table 2).

### Changes in knowledge, attitudes and practices related to antibiotics and their use

The agreement between paired household responses for all variables ranged from no agreement (Cohen’s kappa, κ = -0.003) to weak agreement (κ = 0.22). For example, 12 of the 102 respondents that mentioned an antibiotic during the entry survey could not mention an antibiotic during the exit survey. Similarly, 44 of the 87 respondents who did not consider antibiotics effective against colds/flu during the entry survey considered them effective during the exit survey. In general, the level of agreement was independent of whether the question was about knowledge, attitudes or practices. Nevertheless, the only variables with significant changes in responses between the two surveys were knowledge of an antibiotic, the effectiveness of antibiotics against colds/flu, reliance on previous prescriptions when deciding which antibiotic to buy, always consulting a clinician before taking an antibiotic and trusting nurses for information on the correct use of antibiotics (Wilcoxon test, *P*<0.05; Table 3).

**Table 3:** Total households (counts and proportions; n = 122) in which responses for knowledge, attitude and practice survey questions changed between entry and exit surveys, and the significance levels of the observed changes (Wilcoxon rank sum test, *P* < 0.05).

Sub-group	Variable	HH changed (%)	*P*. value
**Knowledge**	Know or can mention an antibiotic^ŧ^	44 (29.5)	0.003
	Antibiotics effective against colds/flu	52 (42.6)	0.011
	Stop taking antibiotics if feeling okay	21 (17.2)	0.356
	Sharing dose of antibiotics is acceptable	14 (11.5)	0.205
	Remember getting information about antibiotics	58 (47.5)	0.966
	Got information from clinician^Ɨ^	28 (50.0)	0.131
	Got information from health professional^Ɨ^	11 (19.6)	0.493
	Got information from pharmacist^Ɨ^	15 (26.8)	0.753
**Attitude**	Changed view on antibiotics	58 (47.5)	0.966
	Always consult before using antibiotics^Ɨ^	36 (64.3)	0.037
	No taking antibiotics without prescription^Ɨ^	26 (46.4)	0.317
	No self-medication with antibiotics^Ɨ^	23 (41.4)	0.816
	Completing antibiotic doses^Ɨ^	24 (42.9)	0.447
	Trust community pharmacist for information on antibiotics	70 (57.4)	0.369
	Trust clinician for information on antibiotics	46 (37.7)	0.260
	Trust hospital for information on antibiotics	49 (40.2)	0.059
	Trust nurse for information on antibiotics	44 (36.1)	0.009
	Trust health facility for information on antibiotics	10 (8.2)	0.682
**Practice**	Use antibiotics for cough	60 (49.2)	0.803
	Use antibiotics for cold or flu	54 (44.3)	0.239
	Use antibiotics for diarrhea	18 (14.8)	0.692
	Use antibiotics for fever	46 (37.7)	0.764
	Use antibiotics for headache	23 (18.9)	0.911
	Use antibiotic for malaria	15 (12.3)	0.480
	Use antibiotics for pneumonia	15 (12.3)	0.480
	Rely on recommendation from community pharmacist	58 (47.5)	0.606
	Rely on previous prescription by a clinician	31 (25.4)	<0.001
	Rely on own experience on antibiotics	28 (23.0)	0.323
	Rely on opinion of family or friends	11 (9.0)	0.705

^ŧ^Based on 149 household responses (main question)

^Ɨ^based on 56 household responses (sub-question).

**Fig 2:**
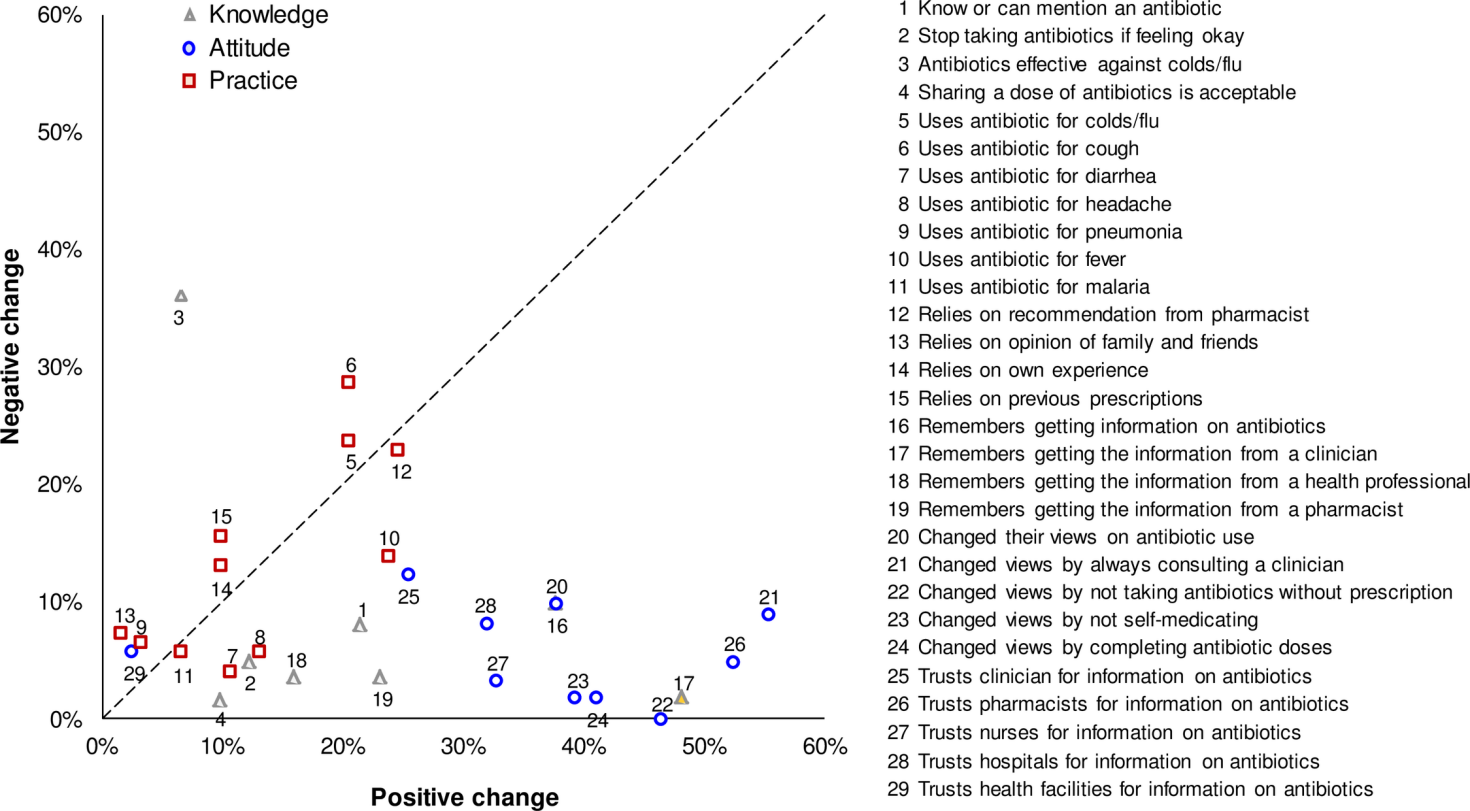
Plot showing the direction of change (entry versus the exit survey) in household responses (n = 122). Twenty-nine knowledge, attitude and practices variables were analyzed. Dashed line depicts no change between entry and exit surveys, variables above the dashed line depict negative change while those below it depict positive change. Variables near the line depict little change while those far from the line depict greater change. Each point represents the proportion of households that changed their responses either negatively of positively during the exit survey compared to the entry survey. Details regarding the determination of the direction of change (positive or negative) are provided in S1 Table.

In general, there was an overall net positive change in households reporting improved knowledge, attitudes and practices with respect to antibiotics (Fig 2). The highest net positive change was for the number of households that considered community pharmacists as trustworthy information sources on antibiotics (32.8%, entry and 80.3%, exit; n = 122). In contrast, the highest net negative change was in the number of respondents who considered antibiotics effective against colds/flu (44.3%, entry and 73.8%, exit, n = 122). Interestingly, changes in knowledge and attitudes (regardless of direction) did not correspond with changes in reported practices. For instance, despite a significant increase in the number of households that considered antibiotics effective against colds/flu (*P* = 0.01), fewer households reported that they would use antibiotics for these conditions (S1 Table), suggesting that knowledge was unlikely to affect practice.

## Discussion

Many studies attribute the increasing prevalence of antimicrobial resistance in sub-Saharan Africa to indiscriminate antibiotic use, exacerbated by uncontrolled access to these therapies [5]. Antibiotic use practices are largely context-specific [2], and several studies in high-income countries have documented more antibiotic prescriptions and use in populations with lower- compared to higher socio-economic status [17–19]. Nevertheless, the patterns of antibiotic resistance at the community level as well as the factors that influence antibiotic use practices in sub-Saharan Africa remain largely undescribed [5]; even less is known about informal settlements.

Our survey of two villages in Kibera, a large informal settlement in Kenya, found that while up to 82% of surveyed respondents mentioned one antibiotic when prompted, very few mentioned a second, while none mentioned a third antibiotic. This suggests either poor antibiotic recall or that respondents were only familiar with a few antibiotics. The two antibiotics that respondents seemed most familiar with were amoxicillin and cotrimoxazole; two broad-spectrum antibiotics that are likely the most widely used in this area, and ones for which several studies in Africa have reported that access is generally ubiquitous [20,21]. Most respondents had partial knowledge on the correct use of antibiotics. For example, they reported that antibiotics should not be shared or that their use should not be discontinued upon alleviation of symptoms. Nevertheless, many considered antibiotics to be effective against colds or flu. While this misconception may be considered an unusual finding for a population that has been the subject of an official infectious disease surveillance program for the past 10 years, studies in different regions of the world report that despite the general population awareness of antibiotics, many people consider these drugs effective against colds and flu [22–24] regardless of evidence in the contrary [25].

Health facilities were the primary sources of antibiotics for the majority of surveyed respondents, with community pharmacists serving as the next most common sources. Several plausible explanations for the higher preference for health facilities exist: (i) this area is served by a study clinic (Tabitha) where PBIDS participants receive care and medications for acute illnesses free-of-charge; (ii) the likelihood of recall bias may have prompted respondents to refer to “Tabitha clinic” as their default source; (iii) the cost implications of privately purchasing antibiotics may have deterred respondents from using alternative sources such as pharmacies; and (iv) misconceptions about the survey objectives (e.g. that surveys would determine whether or not to withdraw the treatment incentive, or whether or not the clinic was an important facility) could have influenced reporting in favor of the study clinic. Other studies have reported important roles for drug dispensers (e.g. community pharmacists, drug hawkers) as antibiotic sources [26] indicating the need for their inclusion in education interventions about antibiotics.

The prevalence of reported antibiotic use in this study was marginally higher than that reported in other parts of the world. For example, out of 12 middle- and low-income countries, 35% to 76% of respondents reported taking antibiotics during the previous six months [27], perhaps consistent with the expected higher burden of disease in an informal settlement. This same report noted that 57% of respondents felt that they could do little to combat antibiotic resistance, which could be a further consequence of a higher burden to disease in these communities. For the current study, colds/flu and coughs were the most common reasons cited by respondents for seeking antibiotics. With the exception of metronidazole, which was only reported for potential use in the treatment of diarrhea, all antibiotics mentioned in the surveys were considered potential treatments for colds/flu and coughs, with or without associated fever. In Kenya, metronidazole is commonly administered as an anti-parasitic drug, particularly for infections caused by *Giardia lamblia* and *Entamoeba histolytica*. It is therefore likely that even when acquired over-the-counter, most households would consider its use primarily for gastrointestinal symptoms that include diarrhea. In general, however, the patterns of reported antibiotic use practices in Kibera differ from those reported in other surveys in Africa. For instance, a study conducted in a Nigerian slum [26] reported significantly higher rates of use of Ampiclox i.e. ampicillin + cloxacillin (79%), tetracycline (54%), metronidazole (51%) and ampicillin (44%). Similarly, a study of antibiotic use in five African counties [2]–including Kenya–showed patterns of use that differ somewhat from those reported herein. In this study, the prevalence of amoxicillin and cotrimoxazole prescriptions was only 37.4 and 27.8% (n = 292), respectively, among Kenyan patients with an acute illness. Differences in study methodologies may account for some of the observed differences. Additionally, Feikin *et al*. [28] found that antibiotic recall declined by 16–23% per week in the two villages we surveyed, and that respondents were less able to recognize antibiotics by name. Both recall and knowledge of antibiotics may have contributed to the variation observed in our study.

Our results show that community pharmacists (who may not be qualified pharmacists) play an important role in influencing household medication-use decisions in this community, consistent with other studies in Africa [6,21]. Close to 70% of respondents reported seeking recommendations from community pharmacists regarding which medicines to buy in the event of an illness. While this contradicts the finding that the majority of respondents cited health facilities as their primary sources of antibiotics, respondents may have been referring more specifically to instances where use of the Tabitha clinic was not feasible or was not deemed necessary. These likely include instances when illness in a household arises beyond the hours of operation of the study clinic, where time constraints limit the use of the clinic or when illness is not considered severe enough to warrant a clinic visit. Nevertheless, the finding that households also relied on previous prescriptions by a clinician and on personal experiences suggests that an unknown degree of self-medication may occur in these communities.

More than half of respondents said they had not received information about the correct use of antibiotics within the last 12 months, which is consistent with reports that antibiotic information is poorly communicated by pharmacies and drug shop operators in sub-Saharan Africa [28]. Given the precipitous rate at which antibiotic recall is reported to decline in this community [29], it is possible that when information is offered, respondents may not accurately recall unless there is significant reinforcement of these messages. Alternately, respondents may have understood the question as referring to information offered through public health education drives, which may be rare in this area.

The finding that respondents consider clinicians, community pharmacists and other healthcare personnel as important and trustworthy sources of information about antibiotics presents an opportunity, not only for providing education on the correct use of antibiotics, but also for correcting the existing misconceptions regarding antibiotics. This can only be realized if physician education about the correct use of antimicrobials is increased. A survey that included 98 resident doctors in a national teaching and referral hospital in Kenya found that only 14.1% of those surveyed had received four or more lectures regarding antibiotic use in the past year [30].

Participation in an observational study may indirectly result in knowledge transfer from the research team to the participant, influencing the outcome of interest. Our evaluation of the potential effect of participant enrollment (into the larger longitudinal study) on household knowledge, attitudes and practices suggests an overall net-positive correlation. Nevertheless, it remains unclear whether unintentional learning occurred among our study participants despite the positive effect. Our analysis revealed low agreement between the responses provided during the entry and exit surveys. This is exemplified by the true-false-response questions where up to 40% of households changed their responses from true to false or *vice-versa* between the two surveys, suggesting that knowledge loss and gain is quite variable in this population. We surmise that knowledge about antibiotics is this community is generally lacking, or superficial at best, and that in the absence of public health education, antibiotic KAP surveys are likely to yield general ideas based on respondents’ value judgements rather than a deeper understanding of the concepts related with the use of antibiotics.

This study probably suffered two common limitations inherent in KAP surveys, i.e., participants providing socially desirable responses, and recall bias. A more unique limitation was that respondents were drawn from an area that has been under health surveillance for a decade, although this ensured our ability to randomly select households and to generally encounter cooperative participants. Thus, the prevalence and types of antibiotics, and in turn, antibiotic use knowledge and practices may have been different from those in other populations. Despite these limitations, the findings from our study contribute to the general knowledge of potential drivers of antibiotic use in informal settlements, which are rarely represented in the literature.

## Conclusion

Our study shows that for the Kibera study population, only 2–3 antibiotics (beta-lactams or sulfa-class antibiotics) are commonly recognized and are probably commonly used in the event of illness within a household, particularly for colds/flu and coughs. The general lack of understanding of what antibiotics are and how they should be used correctly presents a challenge for AMR control efforts. Nevertheless, the existing trust that respondents have in healthcare workers presents an opportunity for targeting educational interventions pertaining to antibiotics and their use, which can over time remedy the prevailing situation.

## Supporting information

S1 File Antibiotic use knowledge, attitudes and practices questionnaire. (PDF)

S1 Table Variables analyzed to show household changes in knowledge, attitudes and practices. (PDF)
