# Supplementary material for: Evidence of superficial knowledge regarding antibiotics and their use: Results of two cross-sectional surveys in an urban informal settlement in Kenya
Source: PLoS One. 2017 Oct 2;12(10):e0185827. doi: 10.1371/journal.pone.0185827 (PMC5624622; doi:10.1371/journal.pone.0185827)
Supplement: S1 File — (PDF) [file pone.0185827.s001.pdf]

S2. Antibiotic Use Knowledge, Attitude and Practices Survey Questionnaire

Knowledge about antibiotics

1. Other than medicines that are used to relieve pain, traditional herbs and medicines that are used to treat malaria, could you mention any commonly used medicines that you know or have heard about ***(GO TO Q.2)***
    - ______________
    - ______________
    - ______________

2. Regarding the medicines you have mentioned (Interviewer to read the antibiotics listed above) please tell me whether you think these statements are "TRUE" or "FALSE". ***(GO TO Q.3)***
    - One should stop taking these medicines as soon as one feels better
    - These medicines are effective against colds and flu
    - It is okay to share these medicines with someone else

Antibiotic use

3. Have you taken any of the medicines you mentioned above within the last 12 months? ***(GO TO Q.4)***
    - Yes
    - No

Ways of obtaining antibiotics

4. In what ways would you obtain these medicines if you felt you needed to use them?
    - Obtain a prescription from a health facility ***(GO TO Q.5)***
    - Without prescription, from a pharmacy ***(GO TO Q.5)***
    - Had some left over from a previous course ***(GO TO Q.5)***
    - Without prescription, from elsewhere ***(GO TO Q.5)***
    - Other – 888 ***(GO TO Q.4.1)***

    4.1. If answered "OTHER", specify how you would obtain antibiotics ***(GO TO Q.5)***

Reasons for taking antibiotics

5. For which illnesses would you normally decide to take the medicines you mentioned above?
    - Cold/flu – 1 ***(GO TO Q.6)***
    - Cough – 2 ***(GO TO Q.6)***
    - Diarrhea – 3 ***(GO TO Q.6)***
    - Fever – 4 ***(GO TO Q.6)***
    - Headache – 5 ***(GO TO Q.6)***
    - Malaria – 6 ***(GO TO Q.6)***
    - Pneumonia – 7 ***(GO TO Q.6)***
    - Skin/wound infection – 8 ***(GO TO Q.6)***
    - Urinary tract infection – 9 ***(GO TO Q.6)***
    - Vomiting – 10 ***(GO TO Q.6)***
    - Other – 888 ***(GO TO Q.5.1)***

    5.1. If "OTHER", specify the illness ***(GO TO Q.6)***

6. How do you normally get information on which to these medicines to buy when you or someone else on the household falls ill?
    - Recommendation by community pharmacists – 1 ***(GO TO Q.7)***
    - Opinion of family or friends – 2 ***(GO TO Q.7)***
    - My own experience – 3 ***(GO TO Q.7)***
    - Previous prescription by a doctor – 4 ***(GO TO Q.7)***
    - Advertisements – 5 ***(GO TO Q.7)***
    - Other – 888 ***(GO TO Q.6.1)***

    6.1. If "OTHER", specify the source of information ***(GO TO Q.7)***

Antibiotic awareness campaigns

7. In the last 12 months, do you remember getting information about the proper use of any of the medicines you mentioned above?
   - Yes ***(GO TO Q.7.1)***
   - No ***(GO TO Q.8)***

7.1. From where did you get information about the proper use of these medicines?
   - Told by doctor ***(GO TO Q.7.2)***
   - Saw it on a TV advertisement ***(GO TO Q.7.2)***
   - Heard it over the radio ***(GO TO Q.7.2)***
   - Read it in newspaper or magazine ***(GO TO Q.7.2)***
   - Told by other health professional (e.g. nurse, community health worker) ***(GO TO Q.7.2)***
   - A friend/family member told me ***(GO TO Q.7.2)***
   - A pharmacist told me ***(GO TO Q.7.2)***
   - Saw it on the internet ***(GO TO Q.7.2)***
   - Other – 888 ***(GO TO Q.7.1.1)***

7.1.1. If "OTHER", specify where they got information from ***(GO TO Q.7.2)***

7.2. What kind of information was this?
   - Information about completing the dosage ***(GO TO Q.7.3)***
   - Information about not sharing this medicine with other people ***(GO TO Q.7.3)***
   - Information about getting the medicine prescribed by a doctor ***(GO TO Q.7.3)***
   - Other – 888 ***(GO TO Q.7.2.1)***

7.2.1. If "OTHER", specify the kind of information ***(GO TO Q.7.3)***

7.3. Did the information you received change your views about such medicines?
   - Yes ***(GO TO Q.7.3.1)***
   - No ***(GO TO Q.8)***

7.3.1. In what ways did this information change your views?
   - You will always consult a doctor in situations where you think you need such medicines ***(GO TO Q.8)***
   - You will no longer take these medicines without a doctor's prescription ***(GO TO Q.8)***
   - You will no longer keep left-over medicines for the next time you are ill ***(GO TO Q.8)***
   - Other – 888 ***(GO TO Q.7.3.2)***

7.3.2. If "OTHER", specify how the respondent's views changed ***(GO TO Q.8)***

Most trustworthy sources of information

8. Supposing you wanted to get trustworthy information about any of the medicines you mentioned above, which sources would you consider seeking information from?
   - A doctor ***(END SURVEY)***
   - A pharmacist ***(END SURVEY)***
   - A nurse ***(END SURVEY)***
   - A hospital ***(END SURVEY)***
   - A health-related internet site ***(END SURVEY)***
   - Family or friends ***(END SURVEY)***
   - Another healthcare facility ***(END SURVEY)***
   - A health-related newspaper or magazine ***(END SURVEY)***
   - Other – 888 ***(GO TO Q.8.1)***

8.1. If "OTHER", specify the source of information ***(END SURVEY)***
