# Supplementary material for: Evidence of superficial knowledge regarding antibiotics and their use: Results of two cross-sectional surveys in an urban informal settlement in Kenya
Source: PLoS One. 2017 Oct 2;12(10):e0185827. doi: 10.1371/journal.pone.0185827 (PMC5624622; doi:10.1371/journal.pone.0185827)
Supplement: S1 Table — (PDF) [file pone.0185827.s002.pdf]

S1 Table. Variables (n=29) analyzed for changes in knowledge, attitude and practices between the entry and exit survey.

Variables	N	Y-Y	N-N	Y-N	Direction (Y-N)	N-Y	% Pos. change	% Neg change	*P.* value
**Knowledge-related**									
Know an antibiotic	149	90	15	12	Neg	**32**	**21.5**	**8.1**	**0.003**
Stop taking antibiotic if feeling better	122	2	99	15	Pos	**6**	**12.3**	**4.9**	**0.356**
Effective for colds/flu	122	46	24	8	Pos	44	6.6	36.1	0.011
Ok to share antibiotic with someone else	122	1	107	12	Pos	2	9.8	1.6	0.205
Remember getting information on antibiotic	122	10	54	12	Neg	46	37.7	9.8	0.966
Remember getting information from doctor	56	6	22	1	Neg	27	48.2	1.8	0.131
Remember getting information from health professional	56	1	44	2	Neg	9	16.1	3.6	0.493
Remember getting information from pharmacist	56	1	40	2	Neg	13	23.2	3.6	0.753
**Attitude-related variables**									
Changed views about antibiotics	122	10	54	12	Neg	46	37.7	9.8	0.966
Always consult a clinician	56	1	19	5	Neg	31	55.4	8.9	0.037
No antibiotic without prescription	56	1	29	0	Neg	26	46.4	0.0	0.317
No self-medication with antibiotic	56	1	32	1	Neg	22	39.3	1.8	0.816
Complete antibiotic doses	56	2	30	1	Neg	23	41.1	1.8	0.447
Trust doctor for antibiotic information	122	64	12	15	Neg	31	25.4	12.3	0.260
Trust pharmacist for antibiotic information	122	34	18	6	Neg	64	52.5	4.9	0.369
Trust nurse for information	122	11	67	4	Neg	40	32.8	3.3	0.009
Trust hospital for antibiotic information	122	16	57	10	Neg	39	32.0	8.2	0.059
Trust health facility for antibiotic information	122	0	112	7	Neg	3	2.5	5.7	0.682
**Practice-related variables**									
Use antibiotic for cold/flu	122	28	40	25	Pos	29	20.5	23.8	0.239
Use antibiotic for cough	122	30	32	25	Pos	35	20.5	28.7	0.803
Use antibiotic for diarrhea	122	1	103	13	Pos	5	10.7	4.1	0.692
Use antibiotic for headache	122	1	98	16	Pos	7	13.1	5.7	0.911
Use antibiotic for pneumonia	122	0	110	4	Pos	8	3.3	6.6	0.603
Use antibiotic for fever	122	6	70	29	Pos	17	23.8	13.9	0.764
Use antibiotic for malaria	122	0	107	8	Pos	7	6.6	5.7	0.480
Recommendation from community pharmacist	122	35	29	28	Neg	30	24.6	23.0	0.606
Rely on opinion of family/friends	122	0	111	2	Pos	9	1.6	7.4	0.705
Use own experience when choosing antibiotic	122	4	90	12	Pos	16	9.8	13.1	0.323
Previous prescription from doctor	122	19	72	12	Pos	19	9.8	15.6	<0.001

Note. N=Number of responses; Y-Y = Yes response at entry and Yes at exit; Y-N = Yes (entry) and No (exit); *P*. value from Wilcoxon rank sum test.
